# Calving duration and obstetric assistance influence pulmonary function of Holstein calves during immediate fetal-to-neonatal transition

**DOI:** 10.1371/journal.pone.0204129

**Published:** 2018-09-18

**Authors:** Camila Infantosi Vannucchi, Liege Cristina Garcia Silva, Silvana Maria Unruh, Cristina Fátima Lúcio, Gisele Almeida Lima Veiga

**Affiliations:** 1 Department of Animal Reproduction, School of Veterinary Medicine and Animal Science, University of São Paulo, São Paulo, São Paulo, Brazil; 2 Radiology and Diagnostic Imaging, Veterinary Hospital, School of Veterinary Medicine and Animal Science, University of São Paulo, São Paulo, São Paulo, Brazil; Van Andel Institute, UNITED STATES

## Abstract

Neonatal immediate adaptation to extrauterine life depends mainly on adequate lung function, which is under the influence of previous fetal maturation and obstetrical condition, both acting to stimulate the efficient liquid removal from the pulmonary parenchyma during the immediate transition period. The objective of the present study is to identify chest radiographic changes of neonatal calves born under the influence of different duration of calving and obstetric assistance and correlate with clinical analysis and blood acid-base balance. Experimental groups were determined according to the duration of calving: 2 h (n  =  16), 2–4 h (n  =  16) and >4 h (n  =  12), and additionally by two sub-groups: no-intervention calving (n  =  22) and intervention calving (n  =  22). Neonatal calves were evaluated for heart and respiratory rate at birth, 5 min, every 10 min until 90 min. Arterial acid-base balance was determined immediately after calving and thoracic radiographs were made at 10 min of life. Lung radiopacity was higher in the 2–4 hr Group compared to the 2 hr Group. When calving duration was greater than 4 hours, a significant respiratory depression was observed. Calving greater than 2 hours slower neonatal pulmonary clearance, 100% and 91.6% of the calves born in the 2–4 hr and >4 hr Groups, respectively, had mild to moderate lung parenchyma opacity. There was a positive correlation between lung radiographic changes and blood TCO_2_ and negative correlation between pulmonary opacity score and blood PaO_2_ and SO_2_. Hence, it is possible to infer that neonatal hypoxia during prolonged calving has an imperative influence on pulmonary fluid absorption in calves. In conclusion, calving greater than 2 hours impacts pulmonary function at birth, leading to altered lung gas exchange, pulmonary clearance, cardiac and respiratory pattern. Conversely, obstetric intervention when calving has duration greater than 4 hours is beneficial for neonatal oxygenation.

## Introduction

Neonatal immediate adaptation to extrauterine life depends mainly on adequate lung functioning, including morphological, physiological and biochemical maturation of the pulmonary parenchyma [1]. During pregnancy, the fetal lung is fulfilled with amniotic fluid, which has to be rapidly absorbed at birth allowing for successful pulmonary gas exchange and oxygenation [2]. The process in which the fluid content is totally removed from the alveoli is called pulmonary clearance, considered essential for the immediate fetal-to-neonatal transition to lung respiration.

The adequate lung functioning of the newborn depends on previous fetal maturation and the obstetrical condition, both acting to stimulate the efficient liquid removal from the pulmonary parenchyma during the transition period [3,4]. In fact, Ramachandrappa and Jain [5] showed that elective C-section (without signs of parturition) increases neonatal risk to respiratory distress in comparison to babies delivered by vaginal labor. Dystocic whelping leaded to slower development of a stable respiratory pattern in neonatal puppies, compared to eutocic vaginal labor or C-section [6]. Therefore, we hypothesize that the duration of calving and obstetric assistance can influence the immediate neonatal pulmonary adaptation to extrauterine life.

In spite of the evident progress in imaging techniques, neonatal radiographic pulmonary assessment is still an important diagnostic tool [7]. Lung radiographic evaluation is of utmost importance for the differential diagnosis of several pulmonary disorders during the neonatal period, frequently employed in human neonatal intensive care units. However, it is important to point out that chest radiography of the newborn has intrinsic peculiarities of the transition period. In fact, Silva et al. [6] showed that 17% of eutocic born puppies had lung radiographic changes, such as pulmonary opacity with little definition of the heart silhouette. Therefore, a high overall interstitial opacity due to low alveolar air content is expected to be found in the radiographic analysis of transition period newborns. In the neonatal calves, lung compliance increases and lung resistance decreases over time, mainly during the first 6 hours after birth [8]. Moreover, complete pulmonary clearance is only achieved within 24 hours of birth [9]. Therefore, radiographic assessment for the purpose of diagnosing pulmonary neonatal disorders should be considered concomitantly with clinical signs and evaluation of blood acid-base imbalance [6]. Nonetheless, a truly radiographic study of the neonatal lung is not frequently performed in the newborn calf, limiting the correlation analysis of radiographic findings with the unspecific clinical signs. Consequently, the precise diagnosis of neonatal pulmonary alterations is not adequately achieved.

The objective of the present study is to identify chest radiographic changes of neonatal calves born under the influence of different duration of calving and obstetric assistance. Moreover, we also aimed to correlate the main lung radiographic findings with clinical analysis and blood acid-base balance.

## Materials and methods

The current study was approved by the Bioethics Committee of the School of Veterinary Medicine and Animal Science—University of São Paulo under the protocol number 1778/2009.

### Animals and experimental groups

As described in detail previously using the same study population [10], the present work was conducted in a single dairy farm located at -21° 54' 14'' latitude, -47° 37' 10'' longitude and 679 m of altitude. The herd was composed of high milk producing Holstein cows and heifers of body condition score between 2.5 and 3.5. Cows were fed a total mixed ration (corn silage and Tifton hay as forage and a corn, soybean, and cottonseed meal-based concentrate) and were provided with ad libitum access to water.

Preparturient heifers and cows were housed individually in a maternity setting approximately one week before they were due to calve, which allowed for an accurate observation of the females. Therefore, calving was constantly observed from the preparatory phase until the complete expulsion of the fetal membranes, always by the same two trained veterinarians. The experimental groups were determined according to the duration of the expulsive phase of calving, understood by the moment of the fetal membranes rupture (allantoid) until the complete expulsion of the calf, as previously described [10]. Therefore, cows were allowed to calve for the following periods:

**2 hours Group** (2 hr; n = 16): expulsive phase of calving with duration lesser than 2 hours. This group was composed of 8 primiparous and 8 pluriparous females, additionally subdivided in No-intervention Calving Sub-Group (females for whom no external obstetrical interference was performed; n = 8) and Intervention Calving Sub-Group (females for whom external obstetrical interference was performed, in equivalent moments to each female of the no-intervention group; n = 8). The mean body weight of calves born within this experimental group was 36.4±4.8 kg.

**2–4 hours Group** (2–4 hr; n = 16): expulsive phase of calving with intermediate duration of 2 to 4 hours. This group was composed of 8 primiparous and 8 pluriparous females, additionally subdivided in No-intervention Calving Sub-Group (n = 8) and Intervention Calving Sub-Group (n = 8). The mean body weight of calves born within this experimental group was 40.2±4.2 kg.

**Greater than 4 hours Group** (> 4 hr; n = 12): expulsive phase of calving with duration higher than 4 hours. This group was composed of 6 primiparous and 6 pluriparous females, additionally subdivided in No-intervention Calving Sub-Group (n = 6) and Intervention Calving Sub-Group (n = 6). The mean body weight of calves born within this experimental group was 38±6.5 kg.

For each cow that calved without assistance (No-intervention Calving Sub-Group), the next parturient female that reached such calving time was subjected to manual extraction of the calf (Intervention Calving Sub-Group), i.e., after the same calving duration between the two subgroups. Thus, we matched exactly the same time elapsed from the rupture of fetal membranes for both experimental sub-groups (No-intervention and Intervention Calving Sub-Group). Forced fetal extraction was considered mild to moderate with the aid of obstetric chains, with tractive force performed by one or two individuals, respectively.

Cows were subjected to external and internal obstetric examination before any manual obstetric intervention. The birth canal was assessed through vaginal palpation, taking into consideration calf malpresentation and overall dilation of the birth canal. Fetal dystocia (malpresentation, twins or oversize calves) or any cause of maternal dystocia (restricted birth canal or uterine inertia) were excluded from the experiment. The extended period of calving in groups 2–4 hr and > 4 hr was attributed to a particular frequency and intensity of uterine contractions presented by some of the subjects, therefore leading to a slower progression of the calf in the birth canal, without being classified as a dystocia.

### Neonatal clinical analysis

Neonatal calves were evaluated for their heart and respiratory rate (through pulmonary auscultation) at birth, 5 min, every 10 min until 90 min. Moreover, we evaluated the respiratory effort (overall respiratory pattern), considering the intensity of the breath sound, regularity of inspiratory movements, presence of intercostal and sternal retractions and presence of crackle lung sound in inspiration and during expiration.

### Blood acid-base analysis

Within 5 min of birth, arterial blood samples (0.5–1 mL) were collected by femoral or coccygeal artery puncture, depending on the accessibility of the blood vessel. Whenever the collection of blood samples from the coccygeal artery was not possible (e.g., low blood pressure at an extremely peripheral artery), arterial blood was collected from the femoral artery. We used 3 mL non-commercial pre-heparinised syringes with 21 G needles (for the femoral artery) or 24 G needles (for the coccygeal artery). After collection, blood was immediately tested using the i-STAT system (Abbott; EG7+ cartridge) for partial pressure of CO2 (PaCO2; mmHg), partial pressure of oxygen (PaO2; mmHg), total carbon dioxide concentration (TCO2; mmol/L), oxygen saturation (sO2; %). All results were corrected for calf body temperature automatically by the analyzer.

### Chest radiographic assessment

Before radiography was taken, all calves were weighted in order to adjust the technique according to calves’ birth weight. Radiography of the lungs was performed 10 min after birth at the peak of inspiration, using three radiographic projections: lateral right-to-left, left-to-right and ventro-dorsal, with the calf in lateral or dorsal recumbency, respectively. A portable X-ray equipment (Pxp 20 HS Plus, Poskom, Poskom Co. Ltd., Goyang, Korea) was used at an exposure of 70–100 kV and 0.8–1.6 mA, according to the birth weight of each calf, and a radiography film 30 x 40 cm T-MAT G/RA (Kodak, Kodak Co. Ltd., São Paulo, SP, Brazil) at a distance of 100 cm between the source and the film. Radiographs were processed in a dark room using Kodak developer and fixer.

**Fig 1:**
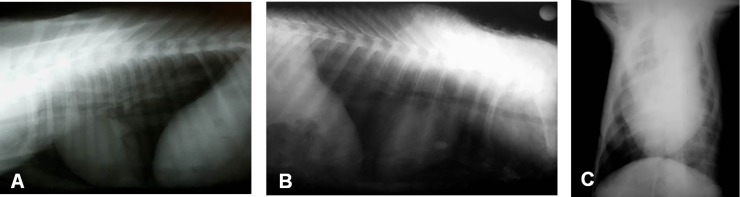
Thoracic radiographic images of neonatal calves. (A) No radiographic alterations–score 0. (B) Mild and diffuse increase in radio-opacity of the caudal lung lobe, with partial indefiniteness of the caudal edge of the cardiac silhouette–score 1. (C) Moderate increase in radio-opacity of the left pulmonary lobe–score 2.

The radiographic analysis was performed in a single-blind manner, by only one experienced veterinary radiologist. Radiographic images were described according to parenchyma opacity of the different lung lobes, graded on an arbitrary scale of 0–3 (Fig 1), being 0 –no radiographic lung alterations; 1- mild degree of diffuse alveolar interstitial opacity of lung fields; 2- moderate degree of diffuse alveolar interstitial opacity of lung fields; 3- severe degree of diffuse alveolar interstitial opacity of lung fields.

### Statistical analysis

The experimental design was a 3 X 2 factorial, considering 3 groups of calving time and 2 groups of obstetrical assistance. All data were evaluated using the SAS System for Windows (SAS Institute Inc., Cary, NC, USA). Differences between treatments were analyzed using parametric and non-parametric tests, according to the residual normality (Gaussian distribution) and variance homogeneity. In order to perform parametric analyses, data were transformed to obey these statistical assumptions. In case no significant interactions were observed, the effect of groups (calving duration) was analyzed merging all sub-groups (obstetric assistance) and conversely, sub-groups were compared combining all groups; otherwise, comparisons were performed taking into account both effects. Differences between treatments were analysed using the LSD Test for multiple comparisons. The response variables were also submitted to Spearman correlation analysis.

Results are reported as untransformed means ± standard error. The significance level was set at 5%. In other words, P-values of ≤0.05 were considered to be statistically significant.

## Results

**Fig 2:**
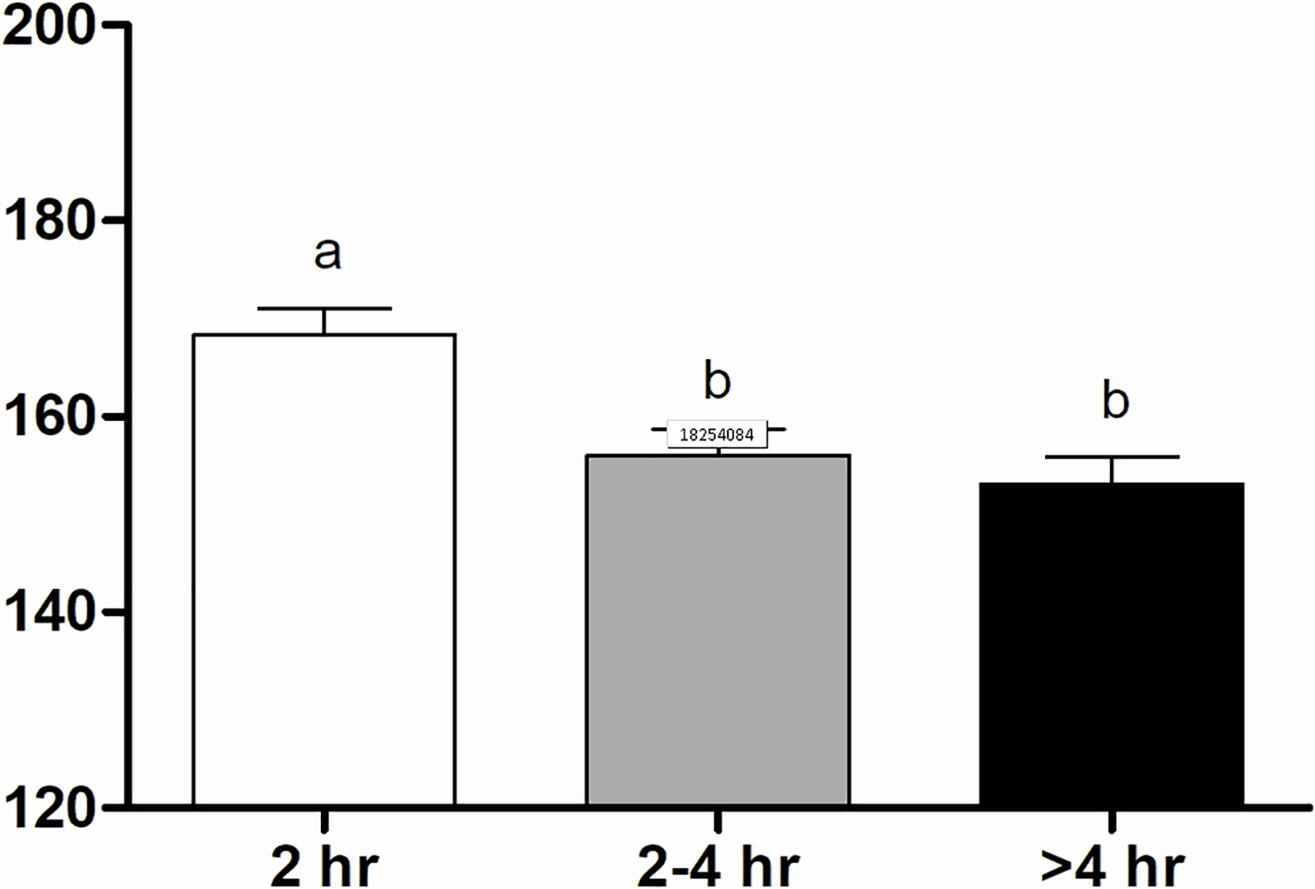
Heart rate (bpm) values of calves in 2 hr, 2–4 hr and > 4 hr groups. ^a-b^ Values with different superscripts differ significantly (P ≤ 0.05).

**Fig 3:**
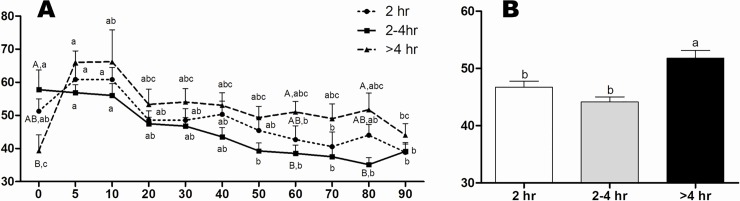
**Respiratory Rate (mpm) overtime (A) of calves in 2 hr, 2–4 hr and > 4 hr groups (B).** ^A-B^ Values with different superscripts differ significantly between groups (P ≤ 0.05). ^a-b^ Values with different superscripts differ significantly with time (P ≤ 0.05).

**Fig 4:**
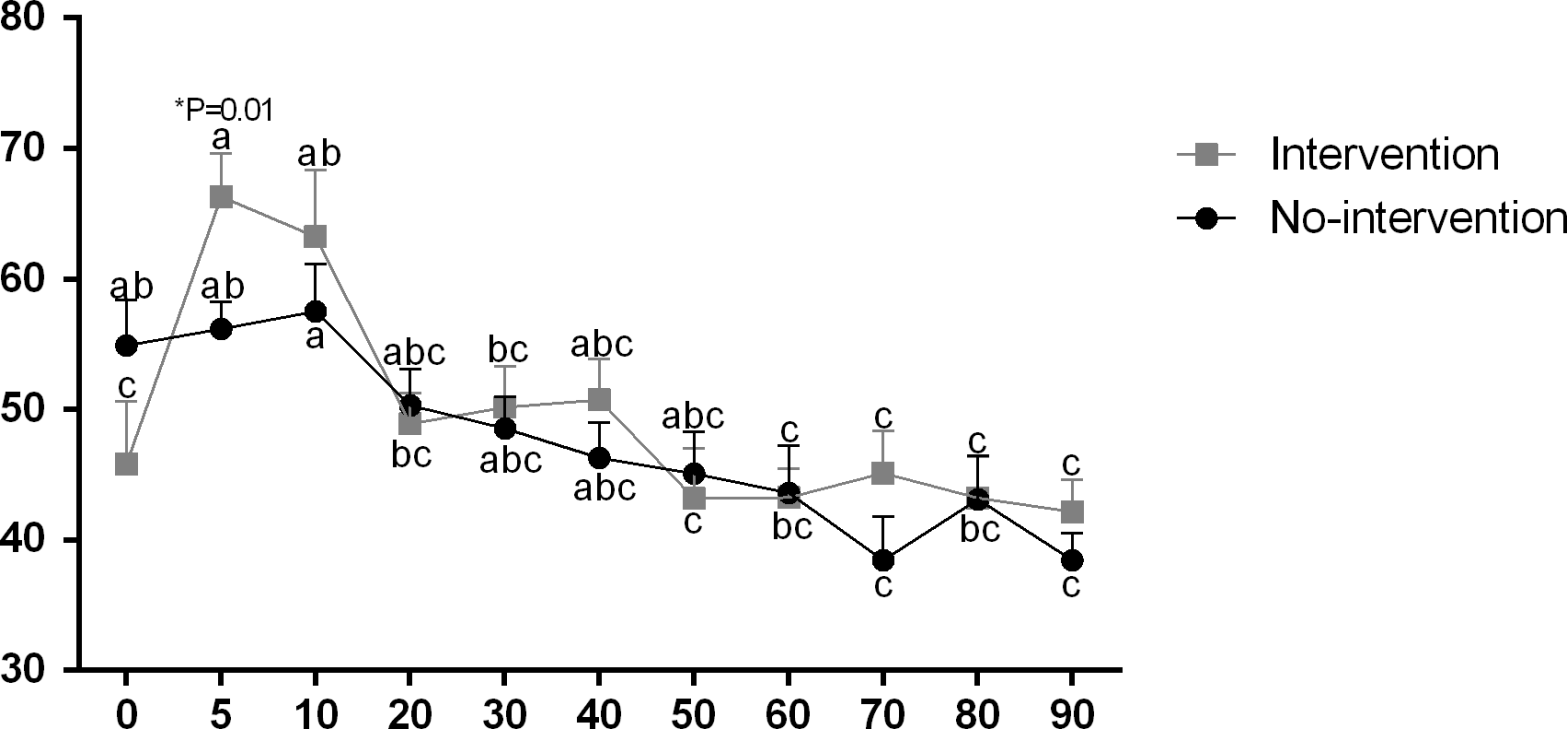
Respiratory Rate (mpm) of calves in No-intervention and Intervention Subgroups. ^a-b^ Values with different superscripts differ significantly with time (P ≤ 0.05). * Significant effect of subgroups within the same time (P = 0.01).

The heart rate of calves born within 2 hr was greater (165±2.3 bpm) than the other groups (155±2.3 e 151±2.4 bpm, respectively for the 2–4 hr and > 4 hr groups) (Fig 2). Moreover, a significant increase in heart rate was observed at 5 min onwards, from which we noticed stabilization. The > 4 hr Group presented lower respiratory rate at birth (39±4.8 mpm) in comparison to the 2–4 hr Group (58±5.9 mpm), however a significant increase in time was verified at 5 and 10 min after calving (66±3.5 mpm and 66±9.6 mpm, respectively; Fig 3A). In addition, calves of the > 4 hr Group had significant tachypnea (52±1.3 mpm) (Fig 3B). Considering the obstetrical assistance, calves respiratory rate from intervention was significantly higher at 5 min (66±3.3 mpm) compared to the No-intervention calving (56±2.0 mpm) (Fig 4). Additionally, a stabilization of the respiratory rate was verified earlier in the Intervention Subgroup (20 min) in comparison to the No-intervention Subgroup (60 min) (Fig 4).

**Fig 5:**
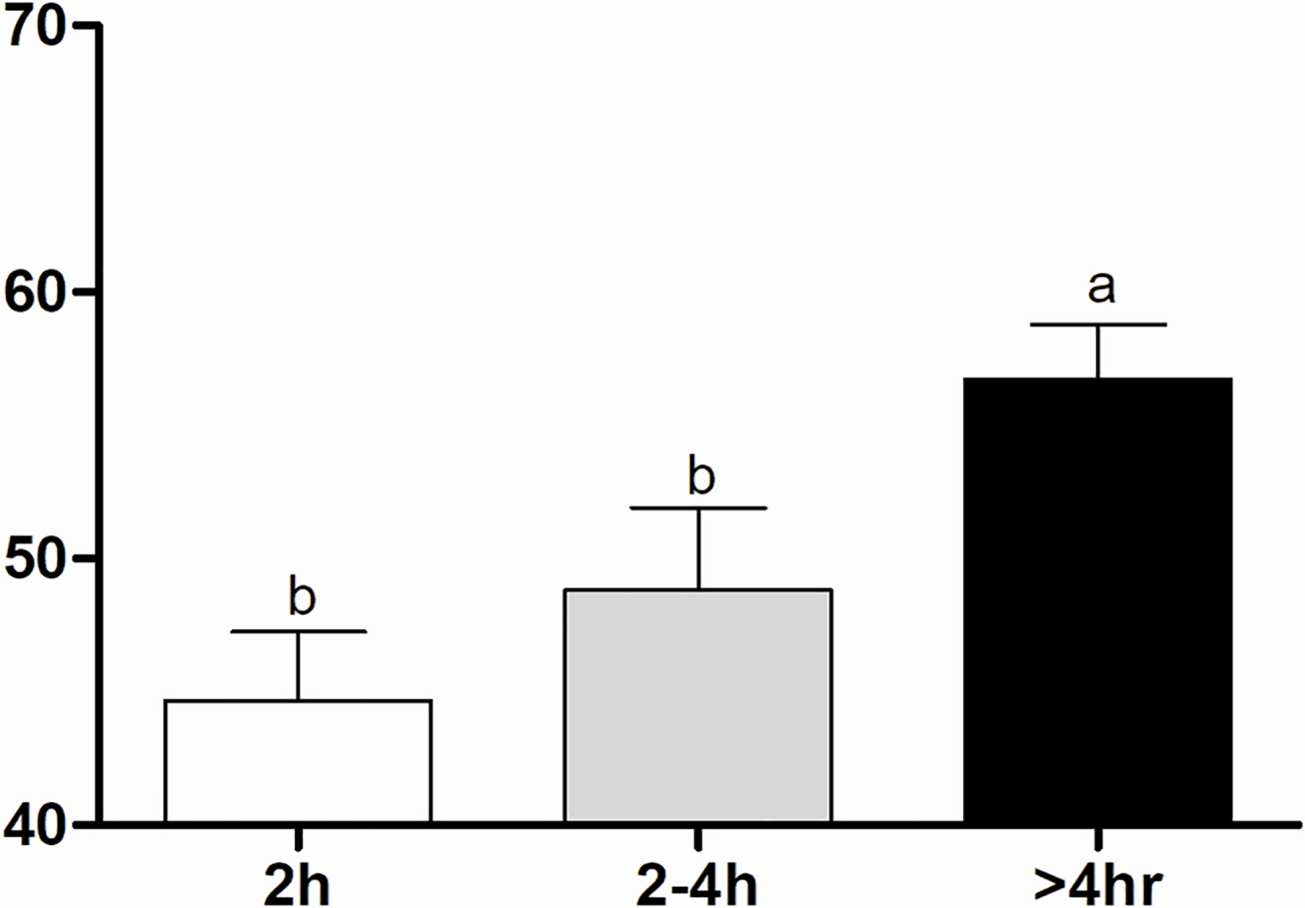
Arterial blood PaCO_2_ (mmHg) of calves in 2 hr, 2–4 hr and > 4 hr groups. ^a-b^ Values with different superscripts differ significantly between groups (P ≤ 0.05).

**Fig 6:**
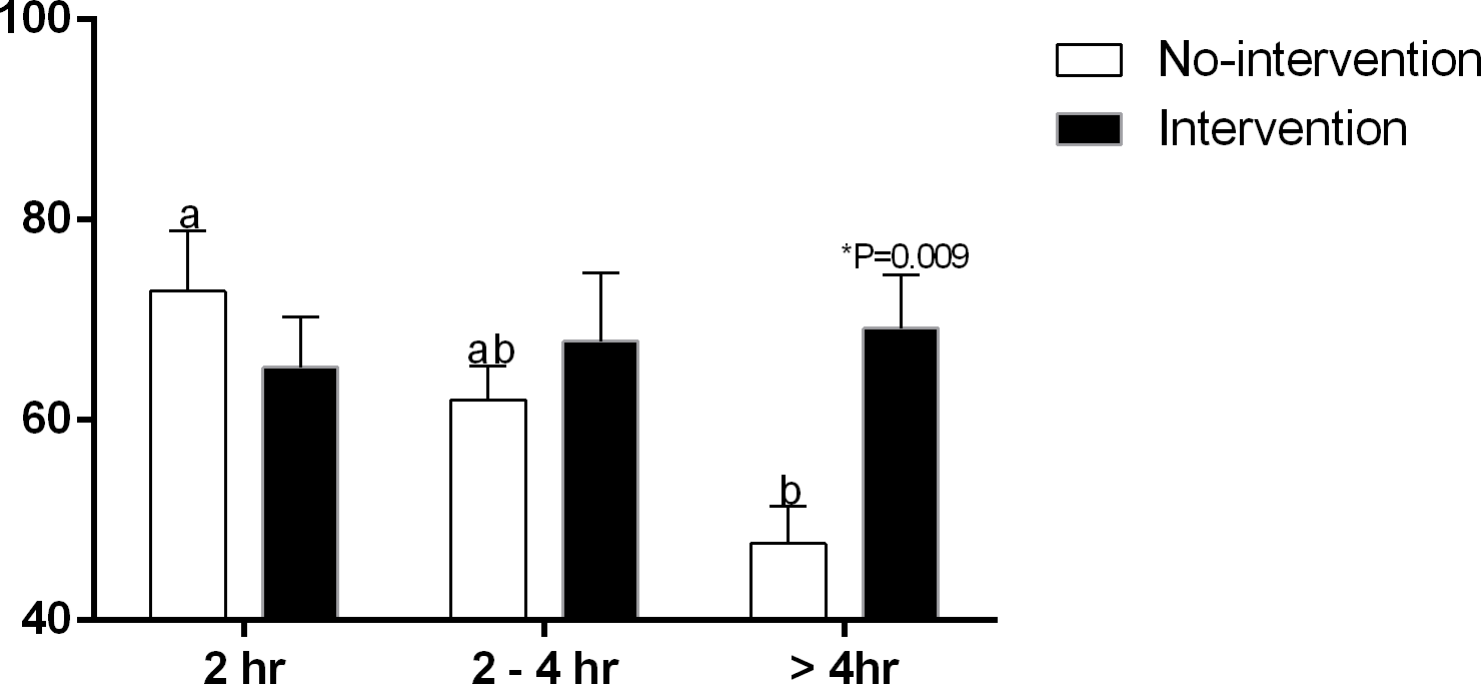
Arterial blood SO_2_ (%) of calves in 2 hr, 2–4 hr and > 4 hr groups and No-intervention and Intervention Subgroups. ^a-b^ Values with different superscripts differ significantly between groups (P ≤ 0.05). * Significant effect of subgroups within the same group (P = 0.009).

The >4 hr Group presented the highest PaCO_2_ at birth (56.7±2.0 mmHg) compared to the 2 hr (44.7±2.6 mmHg) and 2–4 hr groups (48.8±3.1 mmHg) (Fig 5). As calving duration increased, a progressive decrease in oxygen saturation (SO_2_) occurred in calves born without assistance (72.87±5.99, 62±3.39 and 47.66±3.76%, respectively for the 2 hr, 2–4 hr and > 4 hr groups). The > 4 hr Group had the lowest SO_2_ compared to the 2 hr Group (Fig 6). Comparing the obstetrical assistance, > 4 hr calves born by intervention had higher SO_2_ (69.17±5.32%) compared to the No-intervention Subgroup (47.66±3.76%) (Fig 6). The remainder arterial blood gas values did not differ within groups or subgroups (Table 1).

**Table 1:** Arterial blood gas values (means ± standard errors) of calves in 2 hr, 2–4 hr and > 4 hr groups.

	2 hr Group	2–4 hr Group	>4 hr Group	Reference Values
pH	7.26±0.03	7.22±0.04	7.21±0.03	> 7,20 [11]
Bicarbonate (mmol/L)	20.24±1.57	19.71±1.24	21.95±0.93	24,65–30,33 [11]
TCO_2_ (mmol/L)	21.50±1.65	21.13±1.25	23.50±0.91	18–36 [12]
BE (mmol/L)	-3.81±2.29	-7.12±1.65	-5.33±1.37	(-)1,41–5,07 [11]
PaO_2_ (mmHg)	58.69±0.65	50.69±5.15	46.33±3.25	> 70 [13]
K^+1^ (mmol/L)	4.73±0.26	4.70±0.27	4.87±0.32	4,3–6,1 [14]
Na^+1^ (mmol/L)	136.81±0.98	136.94±0.74	136.67±1.04	130–148 [14]

**Fig 7:**
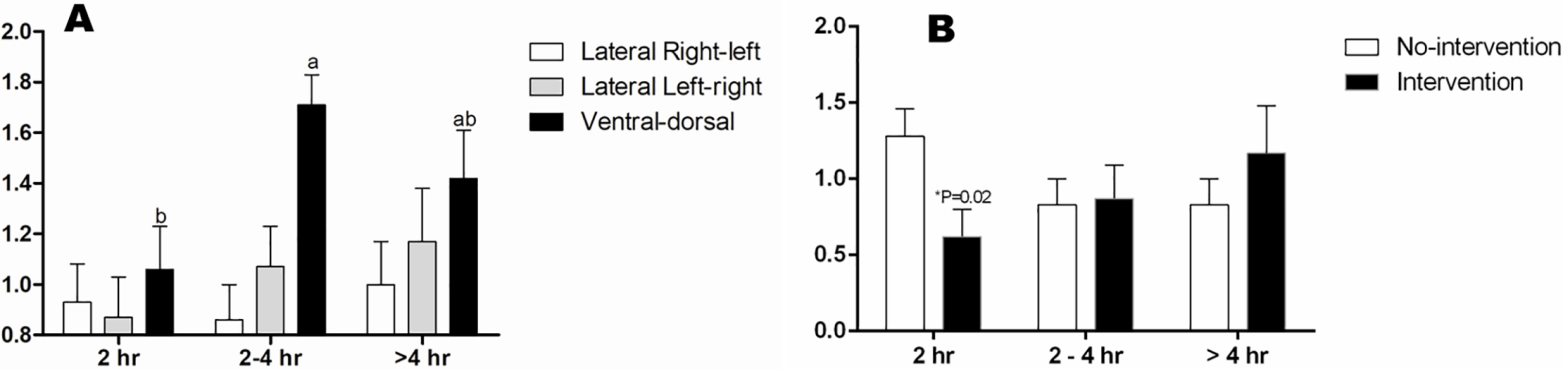
**Pulmonary radiopacity score (0–3) of calves according to the radiographic projection in 2 hr, 2–4 hr and > 4 hr groups (A) and No-intervention and Intervention Subgroups in Lateral Right-left projection (B).** ^a-b^ Values with different superscripts differ significantly between groups (P ≤ 0.05). * Significant effect of subgroups within the same group (P = 0.02).

No neonatal calves presented the severe degree of pulmonary opacity (score 3). The majority of the calves had mild to moderate opacity of pulmonary fields, without any other alterations related to the pleural space and heart. Moreover, on ventral-dorsal projection, hemithorax focal pulmonary opacity was visualized. Among experimental groups, we observed that 87.4% of the calves had mild to moderate pulmonary opacity, whereas 100% and 91.6% of the 2–4 hr and > 4 hr calves, respectively, had radiographic alterations. For the ventro-dorsal projection, the 2–4 hr Group presented higher score of pulmonary opacity compared to the 2 hr Group, not different from the > 4 hr Group (Fig 7A). In addition, for the lateral right-left projection, calves born without assistance within 2 hr had higher score of pulmonary opacity compared to calves born with intervention (Fig 7B).

For the 2 hr calves, we observed a positive correlation between radiographic score on right-to-left projection and TCO_2_ (r = 0.57; p = 0.02), whereas left-to-right and ventro-dorsal projections negatively correlated with PaO_2_ (r = -0.52; p = 0.04) and SO_2_ (r = -0.52; p = 0.04), respectively. The > 4 hr Group presented a negative correlation between left-to-right radiographic projection and SO_2_ (r = -0.65; p = 0.02). Considering the No-intervention calving, there was a positive correlation between the left-to-right radiographic projection and TCO_2_ (r = 0.53; p = 0.02).

## Discussion

The process in which lung fluid is absorbed by transepithelial movement through the alveolar epithelium is named pulmonary clearance and starts at late gestation, extending up to the postnatal period [15]. The pulmonary clearance during the neonatal transition period is of utmost importance for the switch from placental to pulmonary gas exchange [2]. The exact mechanism by which pulmonary epithelium modifies its absorption mechanism is still unknown, but several endogenous factors seem to be involved (steroids, catecholamines, vasopressin and prolactin), as well as mechanical factors such as the alveolar distension by air and exposition to gases [2,15]. However, based on our results, postnatal pulmonary clearance seems to be related to the calving duration, since lung radiopacity score at ventrodorsal projection was higher in the 2–4 hr Group, compared to the 2 hr Group. We assume that prolonged calving can increase fetal hypoxia and hypercapnia, thus triggering the calves’ initial attempts to breathing when still within the birth canal. Therefore, liquid pulmonary content increases before birth, leading to a longer period of time necessary for pulmonary clearance and absorption of alveolar fluid after calving. In fact, when calving duration was greater than 4 hours, a significant respiratory depression was observed at birth, suggesting that respiratory distress is a consequence of higher pulmonary resistance, impeding adequate alveolar expansion by inspired air. Ultimately, decreased alveolar saturation renders a slower reabsorption mechanism by the pulmonary epithelium, delaying lung clearance.

In human babies, the failure to perform pulmonary clearance is the main cause of the Neonatal Transitory Tachypnea (NTT) [16]. Clinically, babies show tachypnea, intercostal retraction, hypercapnia, hypoxemia and respiratory acidosis [2,17]. Chest radiographic analysis shows an interstitial pattern similar to pulmonary edema or irregular opacity with pleural effusion [7]. Based on our results, it is possible to infer that prolonged calving (greater than 2 hours) slower neonatal pulmonary clearance, thus resembling the NTT alteration in human babies. In fact, 100% and 91.6% of the calves born in the 2–4 hr and > 4 hr Groups, respectively, had mild to moderate lung parenchyma opacity. According to Barker and Olver [18], alveolar oxygen has a pivotal role in maintaining the absorptive pattern of the pulmonary epithelium during the postnatal period. Therefore, neonatal hypoxia decreases alveolar clearance, regardless of the degree of pulmonary maturation (i.e., preterm or term neonates) [19]. Indeed, we observed a positive correlation between lung radiographic changes and blood TCO_2_ and negative correlation between pulmonary opacity score and blood PaO_2_ and SO_2_. Hence, it is possible to infer that neonatal hypoxia during prolonged calving (greater than 2 hours) has an imperative influence on pulmonary fluid absorption in calves. Thus, a TTN-like condition can be observed in calves depending on the duration of calving, i.e., calves born within 2 hours are less prone to develop a respiratory distress disease.

Although we could not observe tachypnea and hypercapnia in the 2–4 h Group compared to the 2 h Group, > 4 hr newborn calves had significant higher respiratory rate, hypercapnia and hypoxemia (mainly during No-intervention calving) at birth. Neonatal physiological reflex to moderate or severe acidosis (derived from hypoxemia) is a transitory tachypnea (increased breath per minute and minute volume) with superficial respiratory pattern, trying to compensate the initial acid-base imbalance [8]. In addition, we observed that calving period greater than 2 hours has a depressing cardiogenic effect, noticed by decreased heart rate. Neonatal bradycardia is mainly derived from prolonged hypoxemia, rather than vagal stimuli [20]. Therefore, we believe that calves born from long lasting labor require an increased period of time for pulmonary fluid absorption and complete clearance, ultimately, impairing pulmonary gas exchange and blood oxygenation. We additionally point out that the calving period can influence pulmonary clearance during the neonatal adaptive period and thus compromise cardio-pulmonary function and blood acid-base balance.

Even though prolonged calving negatively impacts neonatal oxygenation, promoting severe hypoxemia and hypercapnia, obstetric intervention favors neonatal oxygenation when calving has duration beyond 4 hours. Interestingly, obstetric intervention in the 2 hr Group resulted in decreased radiographic pulmonary opacity, compared to the No-intervention calving. We suggest that the obstetrical manipulation before 2 hours of calving can provoke increase in catecholamine levels and stressor substances that can, ultimately, favor reabsorption of pulmonary fluid content. However, these results certainly deserve special attention in future studies, by analyzing the influence of different degrees of calving stress on neonatal pulmonary function.

The majority of the calves had pulmonary opacity, frequently restricted to one of the hemithorax (focal). Abdelmegeid et al. [9] showed that complete pulmonary clearance in elective Caesarean-delivered neonatal calves is established only within 24 hours of birth, with thoracic radiographic images compatible with clear lung fields, lung expansion, air content of the lung and absence of lung opacification. On the other hand, both neonatal foals and lambs exhibit clear lung fields by thoracic images up to 6 hours of life, by the time pulmonary pathologic disorders can be diagnosed [21,22]. In human medicine, the main cause for failure in pulmonary liquid reabsorption is the TTN and the Respiratory Distress Syndrome (RDS), which is caused by insufficient surfactant synthesis by the lungs [23]. For RDS babies, radiographic images show mild broncograms without compromising the lung periphery [24]. Therefore, we can assume that radiographic pulmonary opacity in calves immediately after birth is caused by also an inadequate alveolar surfactant synthesis during the perinatal period, leading to interstitial fluid retention. In fact, the alveolar stage of lung development in bovine occurs during the post-natal period [25], which indicates that complete lung alveolarization is established only during the first days of life. Therefore, calves do not have a thorough capacity for pulmonary gas exchange immediately after birth. Thus, pulmonary fluid content negatively affects neonatal vitality and cardiac fulfillment in calves during the transition period.

## Conclusion

In conclusion, calving greater than 2 hours impacts pulmonary function at birth, leading to altered lung gas exchange, pulmonary clearance, cardiac and respiratory pattern. Conversely, obstetric intervention when calving has duration greater than 4 hours is beneficial for neonatal oxygenation.

## Supporting information

S1 File All raw data. (XLSX)
